# *Acheta domesticus* Volvovirus, a Novel Single-Stranded Circular DNA Virus of the House Cricket

**DOI:** 10.1128/genomeA.00079-13

**Published:** 2013-03-14

**Authors:** Hanh T. Pham, Max Bergoin, Peter Tijssen

**Affiliations:** INRS-Institut Armand-Frappier, Laval, QC, Canada

## Abstract

The genome of a novel virus of the house cricket consists of a 2,517-nucleotide (nt) circular single-stranded DNA (ssDNA) molecule with 4 open reading frames (ORFs). One ORF had a low identity to circovirus nucleotide sequences (NS). The unique properties of this volvovirus suggested that it belongs to a new virus family or genus.

## GENOME ANNOUNCEMENT

Cricket-breeding facilities in the United States produce billions of pet-feeder crickets annually (1, 2). The preferred house cricket, *Acheta domesticus*, is highly susceptible to a densovirus, *Acheta domesticus* densovirus (AdDNV), which has caused severe outbreaks since September 2009 and decimated *A. domesticus* stocks in North America. Samples received from die-offs were invariably positive for this virus. However, some recently received samples from mass cricket die-offs in North America were negative for AdDNV.

AdDNV-negative crickets (20 g) were homogenized in 20 ml of a 3:1 mixture of phosphate-buffered saline (PBS) and carbon tetrachloride. After low-speed centrifugation, the upper aqueous phase was passed through 0.45-nm filters and putative viruses were pelleted by centrifugation for 1.5 h at 40,000 rpm and resuspended in a small volume of Tris-EDTA (TE) buffer followed by DNase A and RNase A treatments to remove contaminating nucleic acids. Electron microscopy examination of a 100-fold dilution of the resuspended pellet revealed highly concentrated icosahedral particles of about 18 nm in diameter.

DNA extracted from purified virus by the High Pure viral nucleic acid kit (Roche Applied Science) was resistant to restriction endonucleases and presumably single stranded. Native viral DNA was used for double-stranded DNA synthesis at 30°C by ϕ29 DNA polymerase (3). Amplified DNA was digested with MboI, cloned into the BamHI site of the pBluescriptSK(–) vector, and sequenced by Sanger’s method and primer walking as described before (4). The sequences were assembled by the CAP3 program (5) and generated a 2,517-nucleotide (nt) sequence containing a single EcoRI site. PCR using native DNA and 2 sets of outward primers (with respect to the EcoRI fragment) and sequencing confirmed the circular nature of the genome and the size of 2,517 nt. Due to the circular (rolling) nature of the genome, the name *Acheta domesticus* volvovirus (AdVVV; Volvo [Latin] = roll) was proposed.

Numbering of the genome started with the putative nonanucleotide origin of replication (1-TAGTATTAC), located, as for circo- or cycloviruses (6), between the open reading frames (ORFs) with opposite orientations. Among ORF products of >100 amino acids (aa), ORF1 (361 aa, starting at nt 447) and ORF4 (130 aa, starting at nt 70) were in the sense direction, whereas ORF2 (270 aa, starting at nt 2445) and the overlapping ORF3 (207 aa, starting at nt 2393) were in the antisense direction. BLASTn failed to detect any identity to viral sequences. However, BLASTp revealed a maximum identity of about 30% between ORF2 and Rep proteins of circoviruses and cycloviruses, with a coverage of ~85% (aa 5 to 80, Viral_Rep superfamily [pfam02407], and aa 150 to 212, P-loop_NTPase [pfam00910]). The other ORFs did not have any viral identity using BLASTp.

The lack of sequence identity and the differences in genome organization and size indicated a new virus family or genus. To our knowledge, this is the first circular single-stranded DNA virus in insects that is not related to cycloviruses (7, 8), circoviruses (9–11), nanoviruses (12, 13), or geminiviruses (14, 15), and it may be of interest in elucidating the evolution of this rapidly expanding virus group.

### Nucleotide sequence accession number.

The GenBank accession number for AdVVV is KC543331.
